# Mangrove sediments as carbon sinks: a global analysis of research patterns, trends and future directions

**DOI:** 10.1007/s11356-026-38224-7

**Published:** 2026-09-16

**Authors:** Matheus Cavalcante-Silva, Ana Carolina Santos-Lima, Ana Luisa Fonseca-Oliveira, Vinícius Latgé Meira, Christiane do Nascimento Monte, Wilson Machado

**Affiliations:** 1 Geochemistry Program, Institute of Chemistry, Federal Fluminense University, Outeiro São João Batista, S/N, Centro, Niterói, Rio de Janeiro 24020-140 Brazil; 2 Université d’Angers, Nantes Université, Le Mans Université, CNRS, UMR 6112, Laboratoire de Planétologie Et Géosciences, 49000 Angers, France; 3 Marine Eutrophication Lab (MAREL), CNRS, UERJ, UFF, Rio de Janeiro, Brazil

**Keywords:** Sedimentary organic matter, Blue carbon, Carbon storage, Anthropogenic effects, Organic matter decomposition

## Abstract

Mangrove sediments are widely recognized as an important component of long-term carbon storage in coastal ecosystems. However, the structure, evolution, and thematic organization of the scientific literature addressing mangrove sediment carbon have not yet been clearly characterized at the global scale. This study addresses this gap by providing a scientometric analysis of the literature on mangrove sediments as carbon sinks. Based on 4,798 articles retrieved from the Scopus database, the analysis characterizes publication patterns, major research contributors, and the thematic and collaborative structure of the field. The results indicate an exponential increase in publications across the observed period (R^2^ = 0.9776). A third-order polynomial regression was used exclusively for exploratory projection purposes, indicating a possible 6.7-fold increase in research output over the next two decades (R^2^ = 0.9998). The prominence of the United States and China reflects the consolidation of institutional research capacity and sustained growth in funding and policy support. Trend mapping reveals a historical progression of the field from process-level studies of early diagenesis, through the integration of environmental data, toward a holistic framework in which organic matter degradation is explicitly linked to blue carbon preservation, loss, and dynamics at the ecosystem scale. Overall, this study provides a quantitative synthesis of the evolution and structure of research on mangrove sediment carbon, providing a robust framework to guide future research and inform evidence-based environmental policy.

## Introduction

Anthropogenic activities such as fossil fuel combustion, land-use change, deforestation, forest burning, and industrial development have intensified greenhouse gases (GHG) emissions, particularly carbon dioxide (CO_2_) and methane (CH_4_), driving global climate change (Jackson et al. 2020; Palit et al. 2022; Lin et al. 2024). Although atmospheric CH_4_ concentrations are lower than those of CO_2_, its global warming potential over a 100-year time horizon is substantially higher, reinforcing the relevance of natural ecosystems for climate mitigation strategies (Pachauri et al. 2014; IPCC 2021; Liu et al. 2023; Nisbet et al. 2023). In this context, international frameworks have increasingly emphasized nature-based solutions aimed at enhancing carbon sequestration and long-term storage in natural systems (Macreadie et al. 2019; O’Connor et al. 2020; UNEP 2021; IPCC 2022).

Vegetated coastal ecosystems (VCEs), including mangroves and salt marshes, make a substantial contribution to coastal carbon cycling (Alongi 2020), despite occupying a small fraction of the global coastal area (< 2%) (Herrera-Silveira et al. 2020). Among these systems, mangroves exhibit particularly high carbon burial rates per unit area and are widely recognized as important blue carbon ecosystems (Donato et al. 2011; Hiraishi et al. 2014; Lovelock et al. 2016; Alongi 2018). Blue carbon ecosystems are coastal vegetated environments that capture and store organic carbon over long timescales, and are therefore increasingly recognized as nature-based solutions for climate change mitigation (Nellemann et al. 2009; Duarte and Macreadie 2022; Bandh et al. 2023). In this context, mangroves have been increasingly recognized as strategic ecosystems for climate mitigation and coastal management (Macreadie et al. 2019; O’Connor et al. 2020; Macreadie et al. 2021; Lin et al. 2024).

Recent studies have demonstrated that mangrove sediments can account for between 50% and nearly 100% of the total ecosystem carbon stock and can retain carbon for centuries to millennia, functioning as long-term carbon sinks (Kauffman et al. 2020; Herrera-Silveira et al. 2020; Adame et al. 2021). However, carbon preservation in mangrove sediments is regulated by biogeochemical and biological processes, including microbial decomposition and faunal activity (e.g., bioturbation), which have historically been examined through process-level studies of organic matter degradation during the early diagenesis, but are increasingly investigated within ecosystem-scale frameworks linking carbon storage, GHG dynamics, and climate relevance (Barnes et al. 2006; Kristensen et al. 2008a; Chen et al. 2010; Castillo et al. 2017).

Therefore, the carbon sink function occurs when the rate of carbon input via primary production and burial in the sediments is greater than the rate at which it leaves via export or respiration (Twilley et al. 1992; Breithaupt et al. 2012). Although mangroves contribute a relatively small fraction of global terrestrial carbon sequestration due to a comparatively small global area, their role and potential application in NBS programs is regionally significant, particularly in tropical coastal zones experiencing rapid environmental change (Alongi 2020; Friess et al. 2022).

Despite the growing volume of research addressing mangrove carbon dynamics, the structure, evolution, and thematic organization of the scientific literature specifically focused on mangrove sediments as carbon sinks have not yet been clearly characterized at the global scale. Scientometric analysis provides a quantitative framework for evaluating the intellectual structure and historical evolution of research fields, identifying collaboration patterns and emerging thematic directions through the mapping of scientific outputs (Pritchard 1969; Abad-Segura et al. 2020; Donthu et al. 2021; Herrera-Franco et al. 2021a; Selfe et al. 2025; Colares et al. 2026; Cavalcante-Silva et al. 2026). Although recent scientometric studies have explored blue carbon research across different coastal ecosystems (Huang et al. 2020; Duarte and Macreadie 2022; Jiang et al. 2022; Zhong et al. 2023; Xiang and Cao 2024), analyses explicitly centered on mangrove sediments as carbon sinks remain scarce (e.g., Fest et al. 2022; Xiao et al. 2024).

In this context, this study provides the first global scientometric assessment focused specifically on mangrove sediments as carbon sinks. Based on a bibliometric analysis of 4,798 articles indexed in the Scopus database, the objectives of this research are to: (1) evaluate global publication and citation patterns, (2) analyze the contributions of major research producers, including countries, institutions, funding agencies, journals, and authors, and (3) map collaboration networks and thematic trends to identify research directions and knowledge gaps. By clarifying the structure and evolution of this research field, this study aims to support future scientific investigations and inform evidence-based environmental and climate management strategies.

## Methodology

### Data source, search strategy, and data processing

The methodological framework was structured to directly address the three research objectives defined, combining performance analysis and scientific mapping approaches. The performance analysis is based on the main characteristics of the publications, considering indicators such as the year of publication, number of documents, journals, countries, institutions, funding agencies, authors, and other indicators (Montalván-Burbano et al. 2020; De la Cruz del Río-Rama et al. 2020). Scientific mapping, on the other hand, allows the graphic representation of research fields, to visualize and identify correlations and temporal relations (Cobo et al. 2011; Van Eck and Waltman 2014).

For this purpose, the following methodological criteria were considered: worldwide coverage, quality standards, ease of access, and coverage in the theme addressed in the study (De la Cruz del Río-Rama et al. 2020; Herrera-Franco et al. 2021a). Therefore, it was decided to carry out the study using the Scopus database, considering that this database has a collection with a large number of journals and also more recent publications (Shukla et al. 2019), facilitating data collection.

The chosen Boolean flow was: ("mangrove*" OR "mangrove ecosystem*" OR "mangrove area*" OR "salt marsh*" OR "mangrove forest*" OR "tidal forest*" OR "seagrass*" OR "saline wetland*" OR "tidal wetland*" OR "wetland*") AND ("carbon cycle" OR "carbon sink" OR "carbon sequestration" OR "coastal carbon sequestration" OR "coastal carbon sink" OR "carbon storage" OR "blue carbon" OR "blue carbon system*" OR "carbon stock*" OR "carbon pool" OR "carbon biogeochemistry" OR "carbon geochemistry" OR "mangrove carbon budget" OR "carbon dynamic*" OR "organic carbon cycle" OR "carbon accumulation" OR "carbon loss*") AND ("climatic change*" OR "global warm" OR "greenhouse effect*" OR "dioxide emission*" OR "methane emission*" OR "seasonal change*" OR "seasonal variation*" OR "extreme event*" OR "sea level" OR "environment change*" OR "temperature effect*" OR "climatic phenomena" OR "el nino" OR "la nina" OR "flood*" OR "drought*" OR "tidal export") AND ("mangrove soil*" OR "mangrove sediment*" OR "carbon burial" OR "early diagenesis" OR "sedimentary carbon" OR "pedogenesis" OR "pedogenesis process*" OR "minerali*ation" OR "reminerali*ation").

The descriptors included all fields (such as title, abstract, keywords, title source, affiliations and others). The search was conducted from January 1, 1987, to December 31, 2023, so that all possible references to these topics over more than three and a half decades were included, initially totaling 13,802 documents. After that, some filtering was performed in the Scopus database to ensure the quality of the review.

In this sense, only articles were included, given the rigorous paired review process (Herrera-Franco et al. 2021b). Furthermore, only articles in English were considered, since English is the most widely used language in scientific publications (Cisneros et al. 2018; Herrera-Franco et al. 2020). Finally, the review considered only open access articles, available to the scientific community. A total of 4,798 articles were used in the pre-established bibliometric analysis after filtering.

The bibliographic information of the 4,798 articles was downloaded in a comma-separated values ​​(CSV) file, which is used in several software programs that aim to map scientific productivity (Cobo et al 2011). The original content that should be included in the download includes bibliographic data, such as authors, title, year of publication, source title, author affiliation, keywords, number and citation data, which is extremely important for bibliometric studies (Hallinger and Suriyankietkaew 2018).

### Analysis of publication growth and citation patterns

To address the first research objective, the temporal evolution and relevance of the scientific output were assessed using bibliometric growth indicators. Publication trends were examined based on the annual number of documents, while citation dynamics were analyzed to evaluate the maturity and consolidation of the research field.

The annual growth of publications and citations was first examined using Price’s Law (Price, 1963), which allows the assessment of productivity dynamics and the visualization of literature evolution within a given research area (Montero et al. 2016; Herrera-Franco et al. 2020). In addition, the Price Index was applied as an indicator of research recency, calculated as the proportion of citations from the last five years relative to the total number of citations, multiplied by 100 (Eq. 1) (Wildgaard et al. 2014):

1 $$Price^{\prime}s \;Index = \frac{ number\;of\;citations\;less\;than\;5\;years\;old}{total\;number\;of\;citations} x 100$$

Furthermore, two polynomial regression models were applied in an exploratory and comparative manner to describe the relationship between the cumulative annual number of publications and the year of publication. Previous studies have used third-order polynomials to predict the growth of future publications (e.g., Veiga-del-Baño et al. 2023; Silva et al. 2024; Colares et al. 2026). Similarly, the study by Moshari et al. (2023) used the sixth-order polynomial to predict the growth of publications over the coming decades. Accordingly, both models were compared to evaluate their relative goodness of fit and to characterize publication growth patterns, rather than to produce predictive forecasts.

### Analysis of major research producers

To address the second research objective, the scientific output was examined across different levels of aggregation, including countries, institutions, funding agencies, journals, and authors. This analysis aimed to identify the most productive actors and their relative contributions to the development of research on mangrove sediments as carbon sinks.

Bibliometric data were statistically analyzed and preprocessed using Microsoft Excel to quantify publication and citation indicators associated with each research producer (Herrera-Franco et al. 2020, 2021b). Descriptive productivity metrics were used to compare contributions across spatial, institutional, and disciplinary dimensions, providing an overview of the distribution and concentration of scientific output within the field.

### Collaboration networks and thematic trend mapping

To address the third research objective, scientific mapping techniques were applied to explore the structural and conceptual organization of the literature. This analysis aimed to reveal patterns of scientific collaboration and the emergence of dominant and evolving research themes within the field.

Bibliometric maps were generated using the VOSviewer software developed by Van Eck and Waltman (Van Eck and Waltman 2010), which enables the construction and visualization of bibliometric networks and clustering solutions based on relationships among authors and keywords (Montalván-Burbano et al. 2020). Documents were analyzed in combination, allowing related terms and authors to be grouped into interconnected clusters represented by distinct colors, facilitating the identification and interpretation of collaboration structures and thematic patterns (Van Eck and Waltman 2017). Such mapping approaches have been widely used to visualize research clusters and trends in scientometric studies (e.g., Veiga-del-Baño et al. 2023; Silva et al. 2024; Cavalcante-Silva et al. 2026).

### Limitations and strengths

In this study, the Scopus database was chosen due to its large scale for scientific studies (Baas et al. 2020). However, other database collections that can provide more information on the topic were not included, such as Web of Science. In addition, the choice of Boolean strings should provide comprehensive information on the field of research (Chen et al. 2022), and manual standardization is subject to risks of bias (López-Muñoz et al. 2017). Considering these limitations, this study may underestimate or overestimate the real expressiveness of the results. Nevertheless, the results of this study may contribute to the scientific community by supporting researchers and informing policymakers.

## Results and discussion

### Growth patterns and citation dynamics

**Fig. 1:**
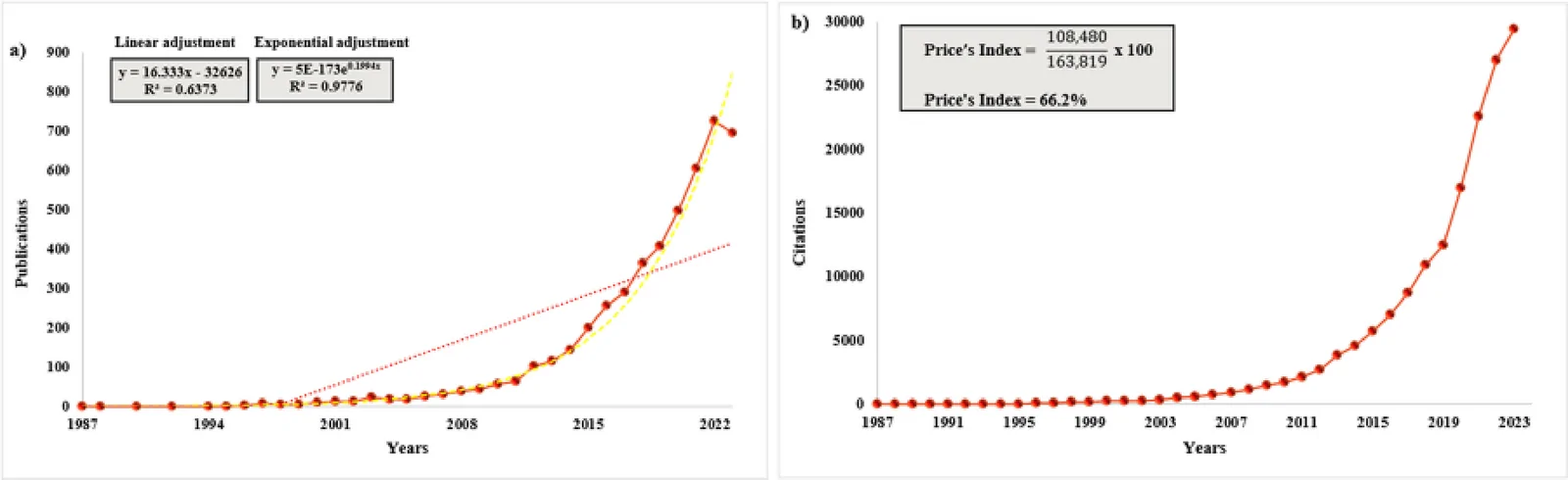
Growth patterns of scientific output and citation dynamics in the research field, showing (**a**) the annual number of publications over time, with linear and exponential model fits used to characterize publication growth patterns, and (**b**) the temporal evolution of annual citations, with research recency assessed using the Price Index

This section examines the temporal evolution and citation dynamics of the research field in order to characterize its overall growth trajectory. Price's Law was used as a bibliometric indicator for productivity analysis, showing exponential growth between 1987–2023. Two types of regression were analyzed, thus obtaining the linear equation *y* = *16,333x—32626* and the exponential equation *y* = *5E-173e*^*0,1994x*^. Given the coefficient of determination (R^2^) of the equations, the exponential growth curve (*R*^*2*^ = *0.9776*) presented a result 1.53 times greater than the linear value (R^2^ = 0.6373), certifying the exponential growth trend of the analyzed data (Fig. 1a).

Price's Law is the most widely used indicator to verify the productivity of a specific research field, in order to reflect the fundamental aspect of scientific production, its exponential growth (Price 1986). The significant increase in research (e.g., Zhao et al. 2020; Hu et al. 2024) reinforces the exponential growth of this theme in the global scientific community.

The productivity analysis verified the relationship between the number of citations per year over the time period pre-established by this study (Fig. 1b). The total number of citations was 163,819, with an average of 4,427.5 citations per year. The year 2023 was the year with the highest number of citations (29,454), followed by 2022 (26,986) and 2021 (22,576). A significant increase was evidenced in the last 10 years (2014–2023), totaling 145,534 citations (88.8%). The Price Index measures the percentage of documents cited in the bibliography in recent years and supports the analysis of the relevance of the topic. If this index is close to 50%, the current literature is very rich (Price 1965). The Price Index obtained by this study was 66.2%, indicating that this area of ​​research is at the core of the scientific community, in constant growth and far from being an obsolete topic.

**Fig. 2:**
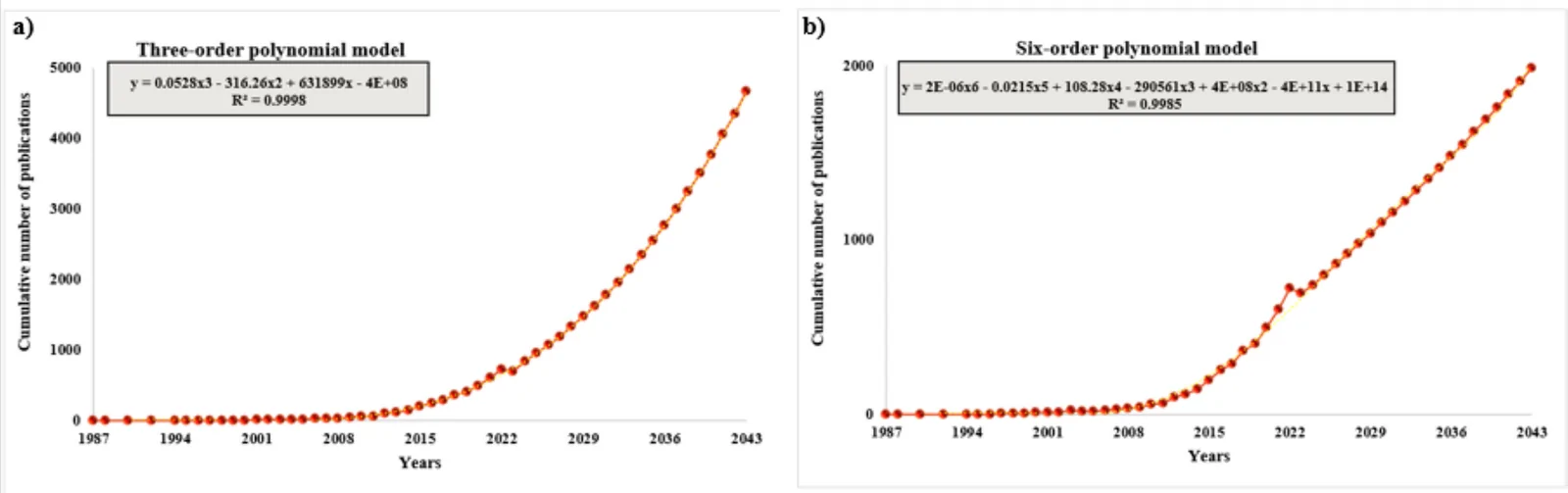
Exploratory polynomial modeling of cumulative publication growth, showing (**a**) a third-order polynomial regression applied to the cumulative number of publications over time, and (**b**) a sixth-order polynomial regression applied to the same dataset for comparative purposes

Exploratory polynomial models were applied to characterize cumulative publication trends, resulting in the following regression fits and coefficients of determination: y = 0.0528 × 3–316.26 × 2 + 631,899 ×—4E + 08 and R^2^ = 0.9998 (third-order polynomial; Fig. 2a); and y = 2E-06x6—0. 0215 × 5 + 108.28 × 4—290,561 × 3 + 4E + 08 × 2—4E + 11x + 1E + 14 and R^2^ = 0.9985 (sixth-order polynomial; Fig. 2b).

These exploratory fits suggest contrasting growth trajectories when extrapolated, with the third-order model indicating cumulative publication levels exceeding ~ 1,400 by 2029 and ~ 4,600 by 2043, whereas the sixth-order model yields more conservative estimates (< 1,050 by 2029 and < 2,000 by 2043). Rather than providing deterministic forecasts, this comparison highlights the sensitivity of long-term growth patterns to model choice and supports the interpretation of a sustained increase in research activity within the field.

Several previous studies have used third-order polynomial models (Veiga-del-Baño et al. 2023; Silva et al. 2024; Colares et al. 2026; Cavalcante-Silva et al. 2026) and sixth-order polynomial models (Moshari et al. 2023) to explore publication growth patterns. Although both models were able to predict significant growth over the next two decades, the third-order polynomial model has a higher R^2^, showing that this model is able to explain around 99.98% of the data variation. On the other hand, on the sixth-order polynomial model, the graph seems to show additional small oscillations, especially around 2023, suggesting that this model may be capturing “noise” or fluctuations in the data, possibly an indication of overfitting (Araujo 2018).

Regarding article citations, the most cited publication was Huguet et al. (2009), which had a citation score of 1.504 (0.9% of the total). This study surpassed the second (Saunois et al. 2019) and third place (Chmura et al. 2003) by 1.12 and 1.16 times, which obtained 1.335 (0.8% of the total) and 1.290 (0.7% of the total) citations, respectively. The ten most cited articles are shown in Table 1. Only 34 articles had more than 500 citations (0.7% of the articles). In total, 4,419 articles had < 100 citations (92.1%).

**Table 1:** Most cited publications on mangrove sediment as carbon sinks

Title	Reference	Journal	Citations
Properties of fluorescent dissolved organic matter in the Gironde Estuary	Huguet et al. (2009)	Organic Geochemistry	1,504
The global methane budget 2000–2017	Saunois et al. (2019)	Earth System Science Data	1,335
Global carbon sequestration in tidal, saline wetland soils	Chmura et al. (2003)	Global Biogeochemical Cycles	1,290
Biogeochemical aspects of atmospheric methane	Cicerone and Oremland (1988)	Global Biogeochemical Cycles	1,202
Major role of marine vegetation on the oceanic carbon cycle	Duarte et al. (2005)	Biogeosciences	1,089
Estimating Global Blue Carbon Emissions from Conversion and Degradation of Vegetated Coastal Ecosystems	Pendleton et al. (2012)	Plos One	1,083
Global patterns of carbon dioxide emissions from soils	Raich and Potter (1995)	Global Biogeochemical Cycles	1,035
Dynamics and distribution of natural and human-caused hypoxia	Rabalais et al. (2010)	Biogeosciences	880
Climatic, edaphic, and biotic controls over storage and turnover of carbon in soils	Schimel et al. (1994)	Global Biogeochemical Cycles	857
Carbon emissions from land use and land-cover change	Houghton et al. (2012)	Biogeosciences	855

### Major research producers and publication structure

This section examines the main research producers and the disciplinary structure of scientific output within the field. The publications were made in 25 major areas of knowledge that address this theme. Considering that the total number of documents has articles in more than one specific area, the total number of records consolidated 9,096 publications. Most of the articles were published in the area of ​​Agricultural and Biological Sciences, with 2,523 articles (27.7% of the total), followed by Environmental Science, with 2,419 articles (26.5% of the total) and Earth and Planetary Science, with 1,781 articles (19.5% of the total).

**Fig. 3:**
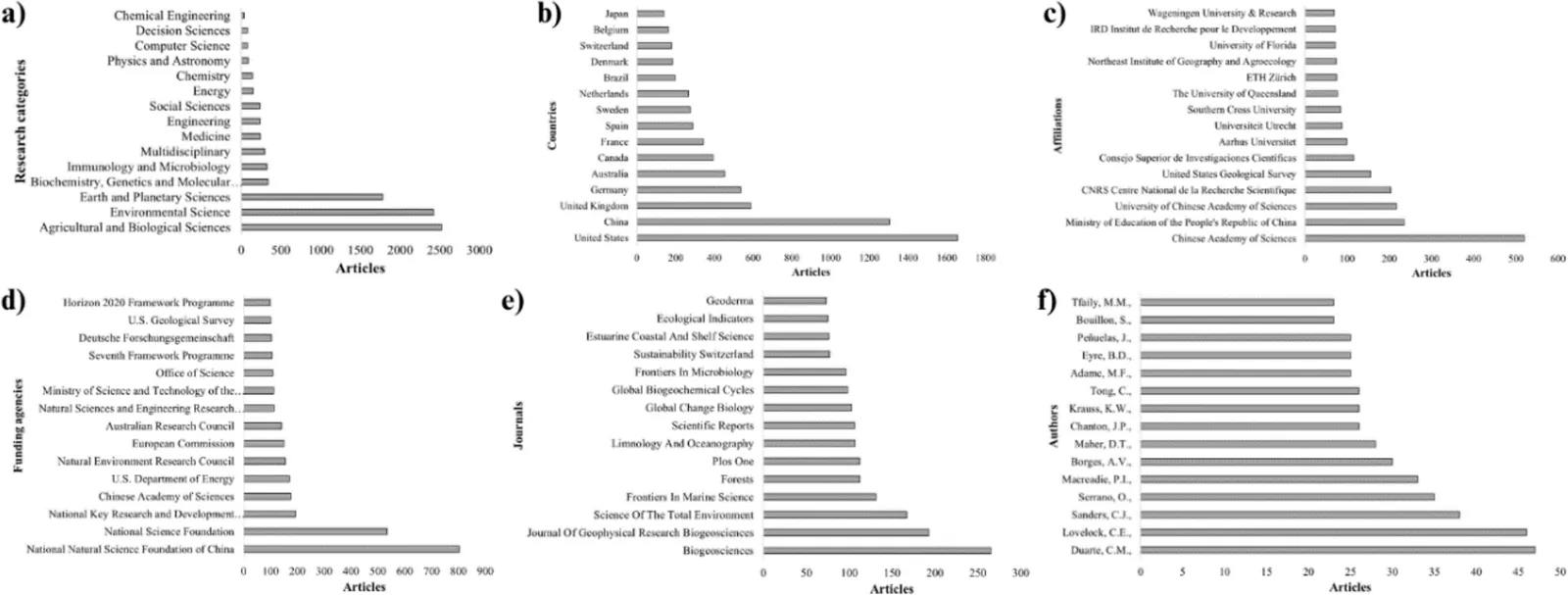
Performance analysis of the main Scopus research categories (**a**), countries (**b**), affiliations (**c**), funding sponsors (**d**), journals (**e**), and authors (**f**) contributing to research on mangrove sediments as carbon sinks

The ten main major areas (Fig. 3a) totaled 8,533 articles (93.81%). This was expected for this topic, considering that climate change demands the dedication of several qualified researchers and analysts who can contribute to the increasingly multidisciplinary scientific challenge to face these changes (Burdon and Zhan 2020).

A total of 144 countries published documents on the subject. Some of the 4,798 articles involved authors from more than one country, resulting in a total of 9,208 country-level records. The USA ranked first, with 1,656 articles (17.9% of publications), followed by China with 1,305 articles (14.1%) (Fig. 3b). The prominent positions of the USA and China were expected, as these countries consistently lead scientific output across multiple fields of knowledge (Andreo-Martínez et al. 2020; Zhou et al. 2022). This prominence likely reflects a dual relevance of mangrove systems in these regions, acting simultaneously as efficient carbon sinks and as vulnerable ecosystems exposed to strong anthropogenic pressures, which has stimulated sustained scientific interest. This interpretation is consistent with patterns reported in previous bibliometric analyses on mangrove research trends (Ho and Mukul 2021) and mangrove-climate change linkages (Segaran et al. 2023).

The prominent contribution of these countries also reflects the increasing alignment between scientific production and climate change mitigation policies, particularly those recognizing mangrove ecosystems as strategic blue carbon reservoirs (Moore et al. 2024). In highly productive and densely populated coastal regions such as those found in the USA and China, mangrove sediments act as long-term carbon sinks through organic matter burial, while being simultaneously exposed to intense anthropogenic pressures, including urbanization and hydrological alteration (e.g., Alongi 2020; Friess et al. 2019).

In total, 168 affiliations were analyzed in this study. Some of the articles had authors with more than one affiliation. Thus, the total number of records for all countries and regions totaled 8,033 publications. The Chinese Academy of Sciences had the highest level of representation, totaling 520 articles (6.4% of publications). The Ministry of Education of the People's Republic of China and the University of the Chinese Academy of Sciences totaled, respectively, 235 articles (2.9% of publications) and 217 articles (2.7% of publications) (Fig. 3c). Several bibliometric studies focusing on mangroves and carbon show a greater contribution from this institution (Ho and Mukul 2021; Jiang et al. 2022; Zhong et al. 2023).

The data on research funding included a total of 159 agencies. Considering that some of the authors had one or more sources of financial contribution, a total of 6,267 articles were counted. The National Natural Science Foundation of China, the National Science Foundation (USA) and the National Research and Development Program of China were the main funding agencies highlighted in this study, with 803 articles (12.8% of publications), 534 articles (8.5% of publications) and 192 articles (3.0% of publications), respectively (Fig. 3d). Sorensen and Jovanović (2021) highlight the importance of affiliations and funding agencies for the productivity of countries and for the standardization of new methodologies and perspectives in this field of study. In this sense, China is expected to show significant growth in the coming years, as it has the three main affiliations and the main funding sponsors.

Approximately 160 different sources were considered in the evaluation of journals. The journal Biogeosciences published the largest number of documents, with 265 articles (5.52% of publications), followed by the Journal of Geophysical Research Biogeosciences (193 articles; 4.0% of publications) and Science of the Total Environment totaling 167 articles (3.4% of publications) (Fig. 3e). These results show great similarity with results obtained by Jiang et al. (2022) and Zhong et al. (2023), since the journals are mainly from the areas of environmental science, biology, geology, atmospheric science, oceanography and other relevant fields.

Approximately 160 distinct authors were identified in the authorship analysis. CM Duarte was the most prolific contributor, authoring or co-authoring 47 publications (0.9% of the total), followed closely by CE Lovelock with 46 publications (0.9%) and CJ Sanders with 38 publications (0.8%) (Fig. 3f). Among these leading authors, CM Duarte is the only one whose work includes a highly influential article directly shaping the conceptual foundations of this research field (Duarte et al. 2005). The prominence of Duarte’s contributions has been consistently emphasized in previous bibliometric assessments addressing carbon dynamics in mangrove ecosystems (Jiang et al. 2022; Zhong et al. 2023) as well as greenhouse gas emissions in marine environments (Chen and Kong 2023).

### Structural and thematic evolution of the research field

The structural and thematic evolution of the research field was explored through scientific mapping techniques, focusing on collaboration patterns among authors and the temporal evolution of research themes derived from keyword analysis.

#### Co-authorship structure and collaboration patterns

**Fig. 4:**
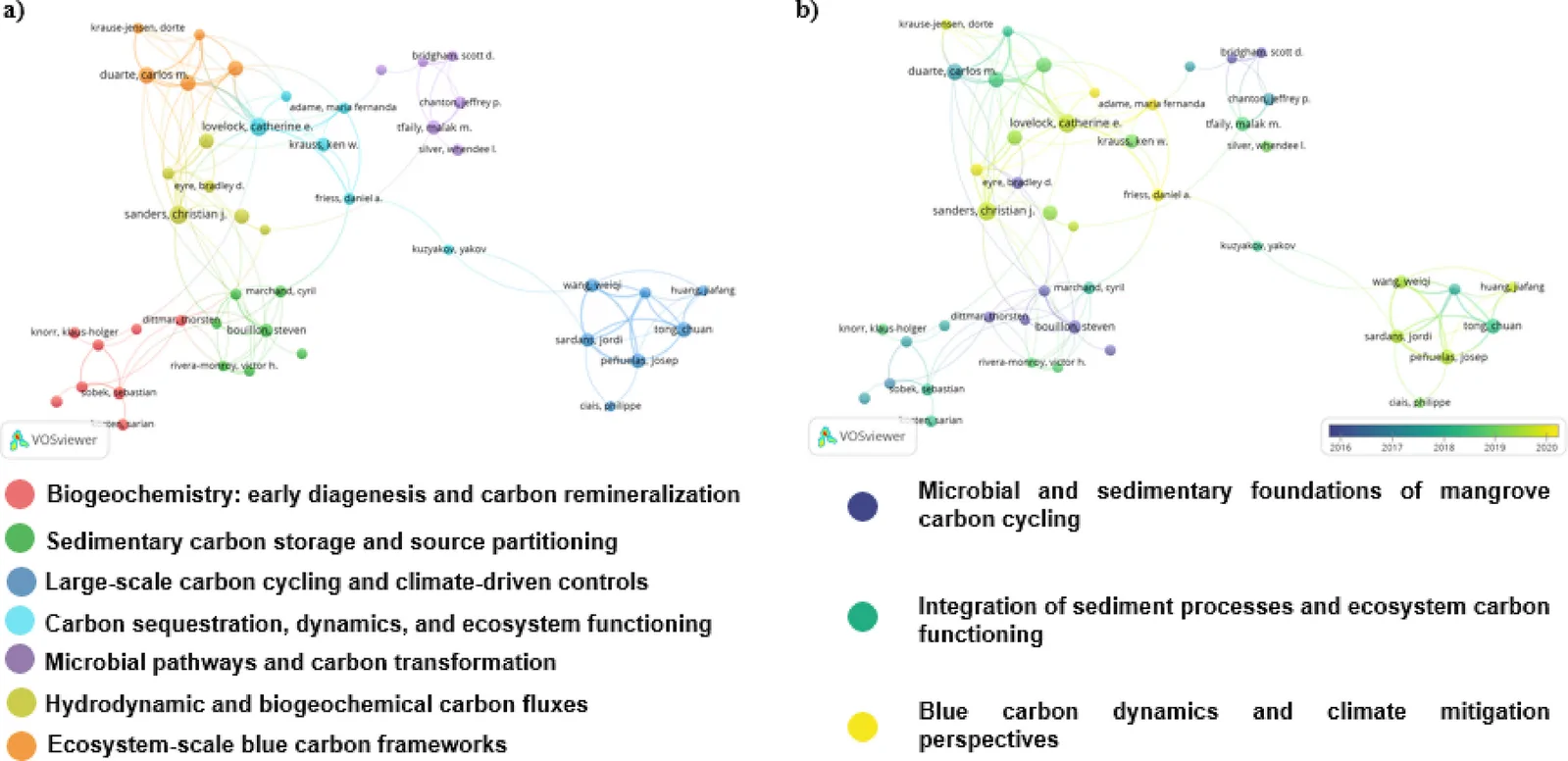
Co-authorship patterns within the literature on mangrove sediments as carbon sinks, with emphasis on the network visualization in (**a**) and the overlay visualization in (**b**). Nodes represent authors, node size reflects publication output, and links indicate co-authorship relationships. In panel (**b**), the color gradient denotes the temporal evolution of publications, showing shifts in collaboration patterns over time

This subsection examines the structure of scientific collaboration within the research field through a co-authorship network analysis. A total of 45 authors were included after filtering the dataset to retain only those connected through at least one co-authorship link, ensuring that the analysis reflects active collaborative relationships. The resulting network reveals seven distinct but interconnected clusters, indicating a highly integrated research field in which thematic communities are linked through shared authorship and cross-cluster collaborations (Fig. 4a).

The cluster “Biogeochemistry: early diagenesis and carbon remineralization” is primarily associated with studies focusing on early diagenetic processes and carbon remineralization under anoxic soil and sediment conditions. This body of work is largely rooted in experimental studies conducted across different ecosystems, including forest soils and freshwater or peatland systems. These studies emphasize microbial degradation pathways, redox-controlled transformations, and the balance between organic carbon preservation and loss (e.g., Dittman et al. 2007; Knorr et al. 2008). This foundational literature provides a mechanistic framework for understanding carbon remineralization, offering an important comparative basis for interpreting biogeochemical processes in mangrove sediments and other coastal wetland systems.

The cluster “Sedimentary carbon storage and source partitioning” encompasses studies that conceptualize mangrove sediments as long-term carbon reservoirs, with a focus on carbon stocks, burial rates, and the relative contributions of organic matter sources. Research within this group commonly integrates stable isotopes, elemental ratios, and sedimentological indicators to discriminate between autochthonous and allochthonous carbon inputs (e.g., Marchand et al. 2006; Kristensen et al. 2008b). Together, these studies establish a process-based link between sedimentary carbon accumulation and the broader framework of carbon storage in mangrove ecosystems.

The cluster “Large-scale carbon cycling and climate-driven controls” reflects a broader perspective on carbon cycling, situating mangrove sediment processes within regional to global biogeochemical and climatic frameworks. Studies in this group emphasize climate-driven controls, cross-ecosystem linkages, and large-scale feedbacks shaping carbon dynamics (e.g., Tian et al. 2015; Huntzinger et al. 2017). Within this context, mangrove sediments are conceptualized as components of interconnected carbon networks that contribute to climate-relevant carbon budgets beyond local sedimentary scales.

The cluster “Carbon sequestration, dynamics and ecosystem functioning” focuses on the functional role of mangrove ecosystems in carbon sequestration, framing the interactions between vegetation, sediment dynamics, and ecosystem functioning. Research within this group examines sediment accretion, root-sediment interactions, and ecosystem resilience as key controls on carbon stabilization (e.g., Lovelock et al. 2016; Buelow et al. 2022). Together, these studies strengthen the link between ecological processes and the capacity of mangrove sediments to function as effective carbon sinks.

The cluster “Microbial pathways and carbon transformation” is characterized by investigations into the microbial mechanisms governing carbon transformation within mangrove sediments. Emphasis is placed on metabolic pathways such as methanogenesis, organic matter decomposition, and molecular-level characterization of carbon compounds (Ogram et al. 2006; Liptzin et al. 2011). These studies elucidate how microbial activity mediates early diagenetic reactions, alters the composition and reactivity of organic matter, and ultimately influences carbon turnover pathways, including both long-term stabilization and gaseous carbon losses.

The cluster “Hydrodynamic and biogeochemical carbon fluxes” focuses on the dynamic exchange of carbon at the sediment–water interface, integrating hydrodynamic transport with coupled biogeochemical processes. Research within this group demonstrates tidal forcing, porewater exchange, and advective transport in regulating the mobilization and redistribution of carbon (e.g., Maher and Eyre 2010; Sanders et al. 2014). Collectively, these studies demonstrate that mangrove sediments function not only as sites of carbon accumulation, but also as active interfaces that mediate lateral carbon export to adjacent aquatic systems.

The cluster “Ecosystem-scale blue carbon frameworks” represents integrative approaches that examine mangrove sediments within the broader functioning of coastal systems, explicitly accounting for the mechanism that influence carbon storage and loss. Research in this group focuses on comparative assessments, sediment-vegetation interactions, and carbon accounting approaches that connect local biogeochemical processes with whole system carbon balances relevant to climate mitigation (e.g., Mazarrasa et al. 2021; Duarte and Macreadie 2022). These studies emphasize mangrove sediments as dynamic components of coastal carbon budgets, governed by process-based controls yet transferable across systems and management contexts.

Beyond the structural organization of collaboration networks, the overlay visualization provides insights into the temporal evolution of research themes within the field (Fig. 4b). By mapping authors according to the average publication year, the overlay reveals a clear progression in scientific focus, linking distinct collaboration clusters to successive phases in the conceptual development of mangrove sediment carbon research.

In the earlier collaborative configuration of the field, research was structured around networks primarily centered on microbial pathways and sedimentary carbon storage. These author clusters were largely grounded in studies of anoxic sediments across a variety of wetland ecosystems, rather than being exclusively focused on mangroves (e.g., Ogram et al. 2006; Marchand et al. 2006; Dittman et al. 2007; Knorr et al. 2008; Kristensen et al. 2008a, b). Within these interconnected research groups, emphasis was placed on mechanistic controls governing carbon transformation, establishing the conceptual and experimental foundations for understanding carbon turnover in anoxic sediments. This collaborative base later supported the expansion toward more mangrove-specific and integrative investigations.

As collaboration networks expanded and became more interconnected, the research focuses progressively shifted toward integrative perspectives linking sedimentary processes to carbon functioning at the coastal system. The cross-cluster interactions in this phase move beyond isolated process descriptions to explicitly consider how biogeochemical reactions, sediment dynamics, and physical transport jointly regulate carbon fluxes, redistribution, and storage across interconnected compartments (e.g., Sanders et al. 2014; Tian et al. 2015; Lovelock et al. 2016). This transition reframes mangrove sediments from discrete reaction zones to dynamically coupled elements of coastal carbon networks, supporting system-level interpretations of mangrove carbon dynamics within coastal carbon budgets.

In more recent collaborative configurations, the field has become increasingly oriented toward climate-relevant and blue carbon frameworks. Expanding international partnerships and cross-disciplinary collaborations now connect sediment biogeochemistry with policy-relevant research on carbon sequestration, restoration, and nature-based solutions (e.g., Friess et al. 2019, 2024; Wang et al. 2021, 2023; Hagger et al. 2024; Twomey et al. 2024). Collectively, these studies explicitly position mangrove sediments within regional to global carbon budgets, underscoring their function as dynamic and management-relevant carbon sinks under changing environmental conditions.

#### Keyword co-occurrence and thematic trends

**Fig. 5:**
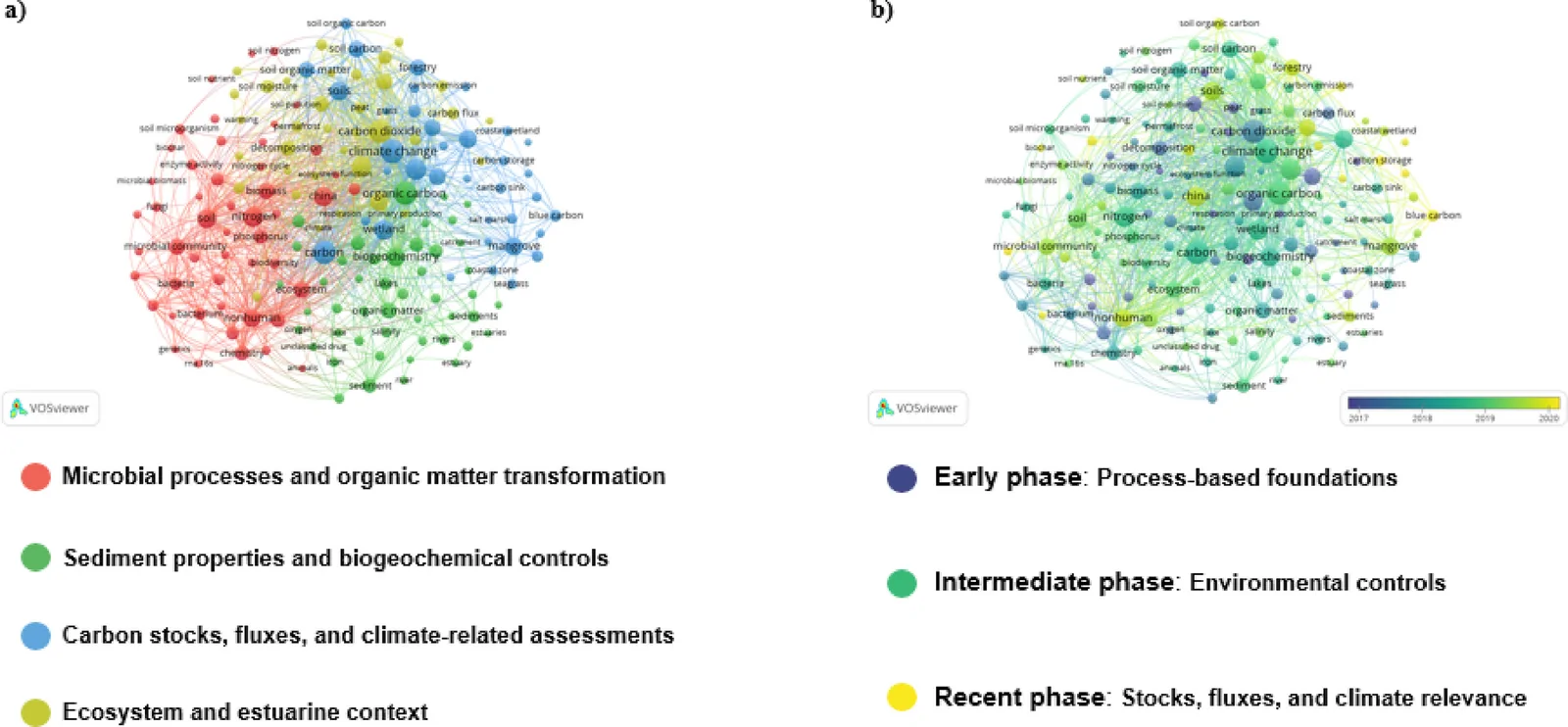
Keyword co-occurrence patterns within the literature on mangrove sediments as carbon sinks, with emphasis on the network visualization in (**a**) and the overlay visualization in (**b**). Nodes represent keywords, node size reflects their frequency of occurrence, and links indicate co-occurrence relationships. In panel (b), the color gradient denotes the temporal evolution of keyword usage, showing shifts in dominant research themes over time

This subsection examines the thematic structure and evolution of the research field based on keyword co-occurrence analysis. To explore the thematic structure of the literature, approximately 250 keywords were considered and grouped into four co-occurrence clusters (Fig. 5a). These clusters define four distinct conceptual networks organizing research on mangrove sediments as carbon sinks.

The cluster “Microbial processes and organic matter transformation” is structured around keywords associated with microbial processes and organic matter transformation, reflecting a body of literature focused on decomposition pathways, microbial communities, and early diagenetic reactions in mangrove sediments. This network emphasizes the role of bacteria-driven processes, enzymatic activity, and organic matter degradation in regulating sedimentary carbon cycling. Studies grouped within this cluster typically address the mechanistic basis of carbon transformation, emphasizing how biological activity governs carbon stabilization or remineralization within mangrove sediments (e.g., Lallier-Vergès et al. 2008; Kauffman et al. 2020; Kim et al. 2021; Chynel et al. 2024).

The cluster “Sediment properties and biogeochemical controls” is linked to sediment characteristics and biogeochemical controls, including sediment composition, nutrient dynamics, and physicochemical conditions influencing carbon preservation. This body of work emphasizes how sedimentary properties such as texture, redox conditions, and nutrient availability regulate organic matter accumulation and transformation. Research within this cluster typically bridges process-level understanding and environmental controls, situating carbon dynamics within the physical and chemical structure of mangrove sediments (e.g., Alongi et al. 2001; Ruiz et al. 2024; Arnaud et al. 2025).

The cluster “Carbon stocks, fluxes, and climate-related frameworks” encompass keywords related to carbon storage, burial rates, CO_2_ fluxes, and blue carbon. This conceptual network reflects research that quantifies sedimentary carbon pools and exchanges, often placing mangrove sediments within broader carbon budgets and climate mitigation contexts. The prominence of climate-oriented terminology indicates a synthesis-oriented literature, in which sedimentary processes are translated into metrics relevant at ecosystem, regional, or global scales (e.g., Murdiyarso et al. 2015; Alongi 2020; Abril et al. 2025).

The cluster “Ecosystem and estuarine context” reflects an estuarine-scale framing of mangrove sediment carbon research. This cluster addresses the ecological context in which sediments operate as carbon sinks, emphasizing the environmental settings that modulate sedimentary carbon processes. Conceptually, it occupies a transitional position between process and stock research, forming a distinct semantic community that links mechanistic studies to ecosystem-scale assessments. Studies within this cluster indicate how landscape setting, ecosystem structure, and land-sea connectivity shape the conditions under which mangrove sediments function as carbon sinks (e.g., Friess et al. 2022; Lovelock et al. 2024, 2025).

The temporal distribution of keywords (Fig. 5b) reveals a clear shift in the thematic focus of the literature on mangrove sediments as carbon sinks. Earlier studies are predominantly associated with process-based approaches, emphasizing microbial activity and biogeochemical transformations governing organic matter degradation. Over time, the prominence of terms related to environmental controls, carbon storage, GHG, and climate relevance increases, indicating a progressive reorientation of the field toward understanding the conditions under which carbon is preserved or released from mangrove sediments.

Within this hotspot trend, the increasing co-occurrence of keywords related to microbial communities, anaerobic metabolism and methanogenesis reflects a shift in the literature toward a more mechanistic framing of microbial controls on sedimentary carbon cycling. At the same time, this body of work increasingly recognizes that many biogeochemical processes operate at the microscale, displaying pronounced variability at millimetric to submillimetric resolutions (Robertson et al. 2008; Kuzyakov and Razavi 2019). Recent studies further emphasize the complexity and context-dependence of microbial metabolic expression in sediments, underscoring the challenges of linking microscale microbial activity to ecosystem-scale carbon dynamics (Kristensen et al. 2017; Bechtold et al. 2025).

While this temporal analysis emphasizes changes in thematic emphasis, the overall importance of specific topics is better captured by their frequency of occurrence across the entire dataset. Accordingly, Table 2 summarizes the ten most frequently occurring keywords together with the number of publications in which they appear. Comparable patterns have been reported in recent bibliometric studies conducted in other ecosystem contexts (Yuan and Sun 2023; Chen et al. 2022; Xu et al. 2023), suggesting that the observed trends reflect broader shifts in environmental and biogeochemical research agendas.

**Table 2:** Most frequent keywords in research on mangrove sediments as carbon sinks

Keyword	Occourrence / Publications (%)	
Climate change	833	17.4%
Organic carbon	802	16.7%
Carbon	800	16.7%
Carbon dioxide	772	16.1%
Wetland	646	13.5%
Methane	621	12.9%
Nitrogen	586	12.2%
China	545	11.4%
Soil	539	11.2%
Biogeochemistry	511	10.7%

This transition reflects a conceptual evolution from classical biogeochemical process studies toward a more integrated perspective centered on the persistence of sedimentary carbon. The increasing co-occurrence of keywords related to “microbial communities” with terms such as “carbon storage” and “climate change” indicates that microbial processes are no longer examined solely in the context of organic matter degradation, but increasingly as regulators of carbon stabilization and long-term storage (Wu et al. 2021; Yuan et al. 2021). In this context, understanding carbon storage requires considering the processes that enable the preservation or loss of organic carbon fractions following degradation, as an integrated outcome of multiple abiotic and biotic constraints acting on sedimentary organic matter (Jennerjahn 2021).

Building on this view, secondary reactions associated with organic matter decomposition, including transformation pathways that influence carbon reactivity and persistence, have become increasingly relevant to sedimentary carbon dynamics. The quantity and stability of organic carbon stored in mangrove sediments are therefore best understood as the outcome of interactions between organic matter sources, composition, and reactivity, modulated by both abiotic constraints (e.g., climate, geomorphology, hydrodynamics, salinity; Sanders et al. 2014; Cacho et al. 2021; Ferreira et al. 2022) and biotic components (e.g., vegetation structure and macrofaunal activity; MacKenzie et al. 2021).

Within this framework, mangrove sediments integrate mixed autochthonous and allochthonous inputs, including plant-derived, soil-derived, and anthropogenic organic matter, whose relative contributions vary across depositional environments and influence carbon preservation pathways (Bauer et al. 2013; Samantaray and Sanyal 2022). Depending on environmental conditions and organic matter quality, these inputs exhibit contrasting degradation trajectories and degrees of preservation (Resmi et al. 2016). This variability underscores the importance of linking organic matter sources to sedimentary processing and burial efficiency.

Sedimentary heterogeneity further modulates these dynamics. Biogenic structures such as crab burrows and root systems alter transport pathways and microscale redox conditions (Thibault de Chanvalon et al. 2017), thereby influencing the preservation and degradation of organic matter in mangrove sediments (Yan et al. 2023). Microbial communities operate within this structured environment, driving organic matter transformation and mediating the balance between remineralization and stabilization. Through a range of aerobic and anaerobic metabolic pathways (including iron, manganese, nitrate, and sulfate reduction, as well as methanogenesis), microbial processes regulate carbon turnover and the formation of secondary compounds that may contribute to longer-term carbon persistence in sediments (Kristensen et al. 2017; Qiu et al. 2023; Booth et al. 2023).

These pathways are further modulated by environmental factors such as tidal inundation, redox conditions, salinity, organic carbon supply, and oxygen availability (Padhy et al. 2020). Anthropogenic pressures, including increased organic matter loading associated with urbanization or aquaculture, can alter these controls by modifying redox conditions and substrate availability, thereby enhancing GHG production and emissions from mangrove and adjacent environments (Martinez-Garcia et al. 2015; Martin et al. 2020; Das et al. 2022; Davenport et al. 2023).

Overall, the growing prominence of keywords related to carbon dioxide, methane, and climate change reflects the translation of mechanistic understanding into ecosystem scale assessments (e.g., Song et al. 2023). Rather than indicating a departure from process-based research, this trend illustrates the maturation of the field, in which insights into organic matter transformation and microbial regulation are increasingly integrated into evaluations of carbon persistence, GHG fluxes, and climate relevance. This integration has also enabled more consistent comparisons of sedimentary carbon dynamics across coastal environments, linking process-level controls to broader depositional contexts.

As sedimentary carbon cycling has increasingly been assessed through integrated metrics linking microbial processes, burial efficiency, and GHG fluxes, mangrove sediments have become more readily comparable to other coastal depositional environments (Alongi 2020; Jennerjahn 2021). This shift has encouraged broader evaluations of how geomorphology, hydrodynamics, and sedimentary regimes influence the long-term fate of organic carbon across contrasting coastal systems (Sanders et al. 2014; Cacho et al. 2021).

In a broader coastal context, mangrove sediments are frequently identified among the most effective coastal carbon sinks. However, their apparent efficiency emerges from contrasts with other depositional environments characterized by distinct hydrodynamic, sedimentary, and biogeochemical regimes. River deltas, coastal lagoons, and coral reef atoll lagoons often display greater variability in long-term carbon storage, reflecting differences in sediment supply, reworking intensity, and organic matter processing (e.g., Feng et al. 2024; Mei et al. 2024).

Quantitative metrics further demonstrate contrasts among these coastal depositional environments, although they are reported in different forms. Mangrove sediments exhibit some of the highest sedimentary carbon stocks, with soil carbon densities commonly ranging from ~ 100 to > 300 Mg C ha^−1^ when integrated over the upper meter (Sanders et al. 2014; Feng et al. 2024). In shallow reef-lagoon systems, carbon stocks are generally lower and confined to shallow sediments, typically spanning ~ 80–280 Mg C ha^−1^, largely controlled by landscape configuration and hydrodynamic exposure (Herrera-Silveira et al. 2025).

River delta wetlands display intermediate but highly heterogeneous carbon stocks, commonly on the order of ~ 50–200 Mg C ha^−1^, shaped by land-use change, sediment redistribution, and hydrological regulation (Chen et al. 2025). By contrast, lagoonal environments are often characterized by variable burial rates rather than large long-term stocks, with organic carbon accumulation increasing from ~ 5–30 to ~ 35–90 g C m^−2^ yr^−1^ under altered hydrological regimes (Mei et al. 2024).

In this sense, mangrove sediments combine high organic carbon inputs with persistent anoxic conditions and effective physical and biogeochemical protection mechanisms, providing a mechanistic explanation for their comparatively high carbon preservation efficiency, even under significant anthropogenic influence (Sanders et al. 2014). This comparison indicates that mangrove carbon sink efficiency arises from the interaction between geomorphology (Arnaud et al. 2025), sediment dynamics (Li et al. 2024), and biogeochemical processes, including microbially mediated decomposition and redox regulation (Booth et al. 2023), beyond the substantial spatial and latitudinal variability observed within and among regions (Ferreira et al. 2022), rather than from carbon input alone.

Representative case studies further illustrate how sedimentary carbon stocks in mangroves vary across geomorphic and anthropogenic gradients. In the Amazon region, mangrove-associated creek and mudflat sediments exhibit high carbon accumulation and storage, with sedimentary carbon stocks commonly exceeding 300 Mg C ha^−1^ when integrated over the upper sediment column (Matos et al. 2020). In Southeast Asia, mangrove sediments show similarly elevated carbon stocks, typically ranging from ~ 360 to 450 Mg C ha^−1^ across protected and unprotected systems, reflecting high organic carbon inputs and efficient sediment burial (Analuddin et al. 2022). By contrast, mangrove sediments in East Africa display more variable and generally lower carbon stocks, commonly on the order of ~ 40–200 Mg C ha^−1^, influenced by sediment lithology, hydrological setting, and intense anthropogenic disturbance in deltaic environments (Nwankwo et al. 2023).

Beyond environmental and anthropogenic controls, variability in reported sedimentary carbon stocks also arises from methodological differences among studies. Carbon stock and accumulation estimates are particularly sensitive to core depth integration, dry bulk density determination, and organic carbon analytical protocols, with shallow cores and assumed bulk density values often leading to underestimation. For example, Sanders et al. (2014) showed that variations in depth integration and bulk density substantially affected calculated carbon accumulation in mangrove sediments, while Cacho et al. (2021) demonstrated that methodological choices combined with local geomorphology strongly influenced organic carbon storage estimates. These examples demonstrate how methodological heterogeneity can bias quantitative comparisons across studies.

## Conclusions

This scientometric assessment provides a comprehensive overview of the structure, evolution, and thematic organization of research on mangrove sediments as carbon sinks. the results demonstrate that this field has experienced sustained exponential growth over recent decades, consistent with Price’s law, with citation patterns indicating increasing scientific relevance. Predictive models suggest that research output is likely to continue expanding in the coming years, reflecting the growing importance of mangrove sediments within the context of carbon cycling and climate change mitigation.

The analysis reveals a highly structured research landscape, characterized by the concentration of scientific output within a limited number of countries, institutions, funding agencies, journals, and leading authors. The prominence of the United States and China reflects strong institutional research capacity, sustained investment in scientific funding, and the consolidation of policy-driven agendas focused on coastal ecosystems and climate-related challenges. This structure is further reinforced by the presence of specialized journals and research programs that have acted as central platforms for advancing mangrove sediment carbon research. At the same time, the diversification of contributing authors and subfields in recent years indicates the expansion and increasing interdisciplinarity of the field.

Keyword and co-authorship thematic mapping reveal a coherent intellectual trajectory in mangrove sediment carbon research, documenting a progression from early process-oriented studies of microbial decomposition and sediment biogeochemistry toward increasingly integrated, ecosystem-scale frameworks. Over time, mechanistic insights into organic matter degradation and early diagenesis have been combined with environmental controls, sedimentary context, and carbon stock assessments, leading to a holistic view that links decomposition processes to blue carbon preservation and loss. Together, these patterns mark a shift from process-level biogeochemistry to ecosystem-scale assessments of blue carbon persistence and climate relevance.

Despite these advances, important conceptual gaps remain. In particular, the literature increasingly emphasizes the complexity and strong context-dependence of microbial metabolic expression in sediments, which poses persistent challenges for linking microscale biogeochemical processes to ecosystem-scale carbon dynamics. In this context, targeted secondary analyses guided by VOSviewer clustering, especially focusing on themes related to microbial communities and methanogenesis, represent a promising avenue for future research. Such focused syntheses would enable deeper abstraction of key findings and unresolved questions within specific thematic hotspots, helping to clarify how microscale microbial heterogeneity propagates to influence blue carbon and GHG dynamics at broader spatial scales.

Overall, this study clarifies the temporal evolution, collaborative structure, and thematic directions of mangrove sediment carbon research and provides a robust framework that guides future investigations, fosters interdisciplinary collaboration, and supports evidence-based environmental and climate decision-making.

## Data Availability

All data generated or analyzed during this study are included in this published article.
